# The Quansys multiplex immunoassay for serum ferritin, C-reactive protein, and α-1-acid glycoprotein showed good comparability with reference-type assays but not for soluble transferrin receptor and retinol-binding protein

**DOI:** 10.1371/journal.pone.0215782

**Published:** 2019-04-29

**Authors:** Razieh Esmaeili, Ming Zhang, Maya R. Sternberg, Carine Mapango, Christine M. Pfeiffer

**Affiliations:** Division of Laboratory Sciences, National Center for Environmental Health, Centers for Disease Control and Prevention, Atlanta, Georgia, United States of America; California State University Fresno, UNITED STATES

## Abstract

The Quansys multiplex (Q-Plex) measures ferritin (Fer), soluble transferrin receptor (sTfR), C-reactive protein (CRP), α-1-acid glycoprotein (AGP), and retinol-binding protein (RBP). We compared Q-Plex results with reference-type assays and evaluated Q-Plex performance. Pearson correlation and Lin’s concordance coefficients between the Q-Plex and reference assay were: Fer 0.98 and 0.91, sTfR 0.88 and 0.35, CRP 0.98 and 0.98, AGP 0.82 and 0.81, and RBP 0.68 and 0.31, respectively. The median relative difference between the Q-Plex and reference assay were: Fer -2.4%, sTfR 107%, CRP 0.03%, AGP -1.3%, and RBP 51%. The Q-Plex intra-assay CVs were <5%; the inter-assay CVs were higher: Fer 11%, sTfR 14%, CRP 9.3%, AGP 7.5%, and RBP 19%. EDTA plasma produced 74% higher Q-Plex sTfR concentrations compared to serum. Analyte stability was good for ≤5 freeze-thaw cycles. After adjusting Q-Plex data to the reference assays, sensitivity and specificity were >85% for Fer and CRP; specificity was >85% for sTfR, AGP, and RBP. Using performance criteria derived from biologic variation, Fer, CRP, and AGP met the minimum allowable imprecision (<10.7%, <31.7%, and <8.5%, respectively) and difference from the reference assay (<±7.7%, <±32.7%, and <±10.3%, respectively), while sTfR and RBP exceeded these thresholds (<8.5% and <7.8% for imprecision and <±7.7% and <±12% for difference, respectively). The Q-Plex measures multiple biomarkers simultaneously, is easy to perform, and uses small sample volumes. With some improvements in accuracy and precision (i.e., sTfR and RBP), this assay could be a useful tool for low-resource laboratories conducting micronutrient surveys for epidemiologic screening applications. These findings need to be verified using other populations, particularly those with inadequate micronutrient status.

## Introduction

Iron and vitamin A deficiency have negative consequences for human health and child development [1,2]. Biomarkers such as serum ferritin (Fer), soluble transferrin receptor (sTfR), and retinol or retinol-binding protein (RBP) are measured to assess population nutritional status. RBP and retinol generally circulate at one to one molar ratio [2]. RBP is sometimes used as a less expensive surrogate for retinol [2]. Inflammation markers such as C-reactive protein (CRP) and α-1-acid glycoprotein (AGP) are also measured to interpret inflammation-dependent nutritional biomarkers [3]. Often adequate laboratory facilities, equipment and supplies, and properly trained staff are lacking to conduct biomarker analyses using widely-accepted laboratory methods and survey samples need to be shipped out of country for analysis, unless there is a restriction for sample export. Furthermore, the analysis of multiple biomarkers by individual assays is costly and requires relatively high sample volumes and each run takes several hours per biomarker (Table 1). Access to reliable, easy-to-use, and affordable assays is needed to accurately assess the population nutritional status in-country.

**Table 1:** Comparison of assay requirements for monoplex vs. multiplex assays^a^.

	Monoplex assay						Multiplex assay
Parameter	Fer	sTfR	CRP	AGP	RBP	Total	Quansys
Approximate specimen volume (μL)	10–20	5–20	2–10	2–10	5–10	24–70	15
Maximum number of samples per plate	40	40	40	40	40	40	40
Approximate time to conduct a run (h)	4–5	4–5	3–5	4–5	4–5	19–25	4–5
Approximate price per sample (US $)	5–13	10–15	12–13	9–12	11–12	47–65	17.5

^a^ Information shown for monoplex assays was derived from at least 3 commercially available ELISA kits.

To address this problem and allow low-and-middle income countries to become self-sufficient in conducting micronutrient surveys, companies have attempted to develop commercially available ‘all-in-one’ instrument platforms that conduct multiple micronutrient tests in a single sample aliquot [4–6]. These instruments need to be inexpensive, of low complexity, and operable by a laboratory technician without requiring specialized training. As importantly, the data produced by these platforms need to be of sufficient quality, reproducibility, and validity for interpretation.

An early version of a multiplex assay for 5 micronutrients (Fer, sTfR, CRP, AGP, and RBP), the Q-Plex Human Micronutrient (5-plex) protein microarray [7], was published in 2014 [6]. In 2017, an expanded version 7-plex including thyroglobulin and HRP2 for malaria was presented [8] and recently the group applied this assay to dried blood spot matrices [9]. The 7-plex microarray was validated against a laboratory-developed test (LDT) sandwich-ELISA from the VitMin Lab [10], which is widely used in micronutrient surveys, using 206 heparinized plasma samples from Nigerian pregnant women [8]. In another comparison of these 2 assays (7-plex and LDT) using 180 serum samples from women and children from Burkina Faso, Cambodia, and Malaysia, poor comparability between the methods was reported [11].

We compared the Quansys 5-plex array, a non-diagnostic research tool, to results obtained with well-established and widely-used clinical commercial assays and for RBP to retinol by HPLC. We consider these particular methods as reference-type assays because they are used as part of the National Health and Nutrition Examination Survey (NHANES) to assess micronutrient status in the US population and/or have been shown to produce result close to the target values of international reference materials. Given that countries often desire to compare their national survey results to NHANES, it is helpful to understand the relationship between the Quansys assay and these CDC assays used in NHANES. Thus, we provide conversion equations from the Q-Plex assay to the reference-type assays. We also evaluated the Q-Plex assay for key method performance parameters and selected pre-analytical factors.

## Materials and methods

### Biological specimens and reference materials

Eighty-five anonymous serum specimens from adult male and female blood donors were purchased from two U.S. commercial blood banks Tennessee Blood Services (Memphis, Tennessee) and BiolVT (Westbury, NY). Of these, 25 specimens had matrix-matched pairs of serum, heparinized plasma (HEP-P) and EDTA plasma (EDTA-P). We obtained the following international reference materials: 94/572 3^rd^ international standard for recombinant ferritin and 07/202 recombinant soluble transferrin receptor reference reagent from the National Institute for Biological Standards and Control (NIBSC); ERM-DA474/IFCC for CRP (spiked) and ERM-DA470k/IFCC for AGP from the European Reference Materials, Institute for Reference Materials and Measurements; and Standard Reference Material (SRM) 968e for retinol from the NIST.

### Methods

The Quansys multiplex assay (Q-Plex), co-developed by PATH (Seattle, WA, USA) and Quansys Biosciences (Logan, UT, USA), is an ELISA-based microarray that simultaneously measures multiple proteins in a single sample aliquot. Q-Plex kits (5-plex array) were purchased from Quansys Biosciences and used according to kit instructions [7] (S1 Text). The manufacturer-specified calibration range (S1 Table) does not necessarily correspond to the reportable range for sample results because actual sample dilution can deviate from recommended 1:10 sample dilution. The Roche cobas 6000 clinical analyzer was used as a reference-type method to compare Fer, sTfR, CRP, and AGP concentrations. Retinol concentrations were measured using a CDC HPLC assay with UV detection.

### Experimentation

#### Accuracy of Q-Plex based on comparison to reference assays

We analyzed 85 serum samples in a single replicate by the 5-plex assay and by the corresponding reference assays (5 runs conducted over 2 weeks).

#### Accuracy of Q-Plex based on international reference materials

We assessed how close the Quansys assay compared to the target values of available reference materials diluted appropriately to produce concentrations within the Quansys calibration range: NIBSC 94/572 and ERM DA-474 1:100 (Fer) and 1:40 (CRP), respectively; other reference materials 1:10 (sTfR, AGP, and retinol). Each diluted sample was analyzed in duplicate in 1 run.

#### Imprecision of Q-Plex

We assessed the Q-Plex intra-assay imprecision by analyzing 4 serum samples in 5 replicates in 1 run and the inter-assay imprecision by using the same 4 samples analyzed as a single replicate in 10 runs.

#### Effect of pre-analytical factors on Q-Plex results

We assessed the effect of selected pre-analytical factors on Q-Plex results (S2 Text): dilution linearity at 1:5, 1:20, and 1:40 compared to manufacturer recommended 1:10 dilution (*n* = 5); comparison of matrix-matched pairs of serum, HEP-P, and EDTA-P samples (*n* = 25 per matrix; also conducted for reference assays); stability for ≤5 freeze-thaw cycles for serum, HEP-P, and EDTA-P (*n* = 5 samples per matrix); and effect of elevated (30°C) plate incubation temperature for serum, HEP-P, and EDTA-P (*n*~35–40 samples) compared to manufacturer recommended 20–25°C.

### Statistical analysis

Due to assumption violations (non-constant difference and non-constant variance) with the standard Bland-Altman limits of agreement (LoA) method, we employed a variation of the LoA method to assess agreement between 2 assays (S3 Text). We used linear regression of the difference between the assays on the average of the assays to derive prediction equations from the test assay to the reference assay [12]. Where appropriate (i.e., Fer, sTfR, CRP, and RBP), we log (natural) transformed the data to address the assumption violations. In these cases, after back-transforming the model estimates, the relationship between the 2 assays and the prediction limits were non-linear. We used the prediction equations to calculate predicted values and 95% prediction intervals at selected measured values (minimum, 25^th^, 50^th^, and 75^th^ percentiles, and maximum as measured by the reference assay). To minimize the impact of influential points on the prediction equations and subsequent analyses, we removed 4 data pairs (Fer: n = 2; CRP n = 1, sTfR: n = 1). The criterion for removal was based on the calculated Cook’s D (distance) for each data value from a regression of the difference between the assays on the average of the assays. Cook’s D measures the effect of omitting the data pair on the estimated regression coefficients. The Cook’s D of each of the data pairs removed from the analysis all exceeded 5 times the traditional cutoff for Cook’s D (4/n) A total of 77, 72, 83, 85, and 85 serum samples were used in this analysis for Fer, CRP, sTfR, AGP, and RBP.

Additionally, we used a non-parametric approach to describe the agreement between the 2 methods by reporting the proportion of the relative differences that fall within selected limits (e.g., within ±5% of the reference assay). *P* values ≤0.05 were considered statistically significant.

To assess the Q-Plex assay difference to each reference assay, we calculated the median relative difference across serum samples. We assessed the acceptability of the difference by comparing to the minimum allowable difference based on biologic variation: difference = 0.375*(within-individual CV^2^+between-individual CV^2^)^1/2^ [13]. For objective quality goals for method performance, see S2 Table.

We evaluated the diagnostic characteristics of the Q-Plex assay by calculating sensitivity, specificity, positive predictive value (PPV), and negative predictive value (NPV) using results from the reference assay as a gold-standard and commonly used cutoff values to define deficiency or inflammation: <15 μg/L for Fer [1], >5.3 mg/L for sTfR [14], >5 mg/L for CRP [3], >1 g/L for AGP [3], and <0.7 μmol/L for RBP [2].

To assess the Q-Plex assay imprecision, we calculated the mean CV across 4 serum samples for 5 replicates measured in 1 run (intra-assay CV) and for 1 replicate measured in 10 runs (inter-assay CV). The acceptability of the imprecision was judged by comparing to the minimum allowable imprecision based on biologic variation: analytical CV = 0.75*within-individual CV [13].

## Results

### Accuracy and diagnostic characteristics of Q-Plex based on comparison to reference assays

The concentration range of the 85 serum samples as measured by the reference assays covered normal and abnormal values, except for retinol, where none of the samples had low retinol concentrations <0.7 μmol/L (Table 2). For Fer, sTfR, and CRP we had incomplete sample sets. The Q-Plex assay had 5, 1, and 12 no reportable results for Fer, sTfR, and CRP, respectively. Furthermore, we excluded 4 outliers (Quansys/Roche): Fer (1.8/13.6 and 505/376 μg/L); sTfR (70.5/14.4 mg/L); and CRP (141/52 mg/L).

**Table 2:** Agreement between Q-Plex and reference assay for serum samples^a^.

Parameter	Fer (μg/L)	sTfR (mg/L)	CRP (mg/L)	AGP (g/L)	RBP (μmol/L)
Sample size^b^, *n*	78	83	72	85	85
Influential points excluded, *n*	2	1	1	0	0
Concentration range^c^	6.8–288.1	2.1–15.9	0.3–23.0	0.5–1.5	0.8–2.7
Non-constant difference^d^					
Original data *P* value	<0.0001	<0.0001	<0.0001	0.023	<0.0001
Log-data *P* value	<0.0001	0.054	0.18	0.028	0.0002
Non-constant variance^e^					
Original data *P* value	<0.0001	<0.0001	<0.0001	0.08	0.0127
Log-data *P* value	0.51	0.52	0.96	0.045	0.08
Natural log transformation applied^f^	yes	yes	yes	no	yes
Pearson correlation *r*	0.98	0.88	0.98	0.82	0.68
Lin’s concordance *rho* (95% CI)	0.91 (0.89, 0.93)	0.35 (0.27, 0.43)	0.98 (0.97, 0.99)	0.81 (0.73, 0.87)	0.31 (0.21, 0.4)
Mean difference to reference (SD)	-0.15 (0.423)	0.72 (0.20)	0.035 (0.182)	-0.017 (0.123)	0.443 (0.252)
Median relative difference (IQR) to reference, %	-2.4 (-32.8, 15.2)	107 (87, 141)	0.03 (-8.6, 16)	-1.3 (-9.9, 7.6)	51 (24, 86)
Minimum allowable relative difference, %	±7.7	±7.7	±33	±10	±12

^a^ AGP, α-1-acid glycoprotein; CRP, C-reactive protein; Fer, ferritin; RBP, retinol-binding protein; sTfR, soluble transferrin receptor; Roche clinical analyzer assays used as reference assays for Fer, sTfR, CRP, and AGP; retinol measured by HPLC used as reference assay for RBP

^b^ *n* = 85, unless removals caused by missing (no reportable) value, outlier, or out of range sample(s)

^c^ As measured by reference assay after exclusion of outlier or out of range sample(s)

^d^ The *P* value tests the null hypothesis that the slope coefficient is zero from a regression of the differences on the averages

^e^ The *P* value tests the null hypothesis that the slope coefficient is zero from a regression of the absolute residuals on the averages, where the residuals are computed from a regression of the difference on the averages

^f^ Data analysis performed and reported on natural log scale (Pearson correlation, Lin’s concordance, mean difference to reference

Difference plots of the original data (S1 Fig, panels A-E) showed non-constant variance and non-constant difference for Fer (panel A), sTfR (panel B), CRP (panel C), and RBP (panel E); AGP showed constant variance and non-constant difference (panel D). Difference plots of the log-transformed data (S1 Fig, panels F-J) showed constant variance and constant difference for sTfR (panel G) and CRP (panel H); however, Fer (panel F) and RBP (panel J) still showed non-constant difference. No log-transformation was necessary for AGP. The visual interpretation was supported by the *P* values for non-constant variance and non-constant difference derived from the original and log-data models (Table 2).

We observed high Pearson correlation coefficients between the Quansys and the reference assay for Fer (*r* = 0.98), CRP (*r* = 0.98), sTfR (*r* = 0.88), and AGP (*r* = 0.82) and moderate correlation between RBP and retinol (*r* = 0.68) (Table 2). The Lin’s concordance coefficient was also high for Fer (*rho* = 0.91), CRP (*rho* = 0.98), and AGP (*rho* = 0.81), but much lower for sTfR (*rho* = 0.35) and RBP (*rho* = 0.31). The discrepancy between these 2 coefficients suggests that while the 2 methods have a strong linear relationship there is poorer agreement between the 2 methods.

**Fig 1:**
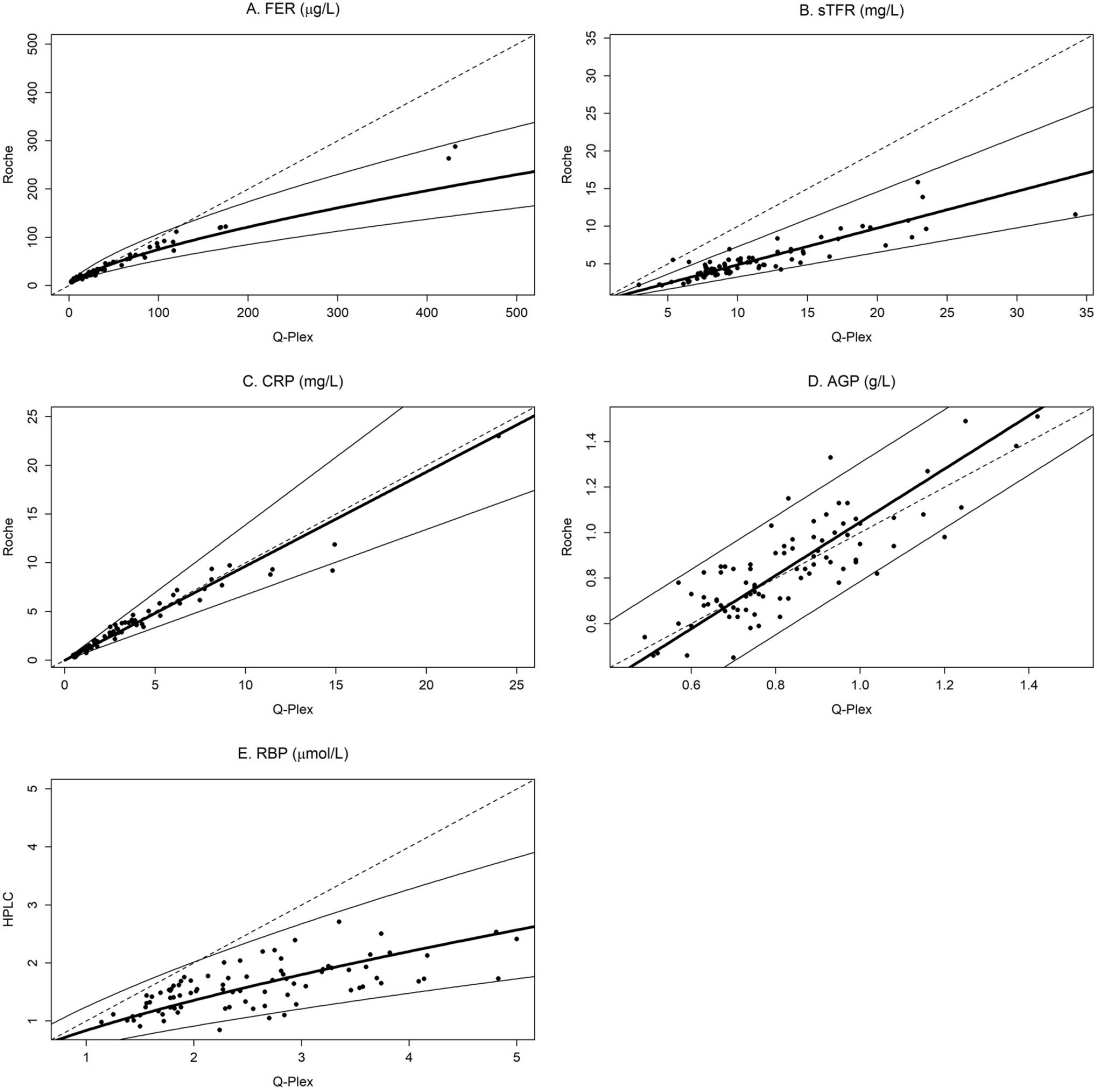
Prediction plots of the test assay on the y-axis and the reference assay on the x-axis showing the prediction line and 95% prediction intervals. The prediction lines and 95% limits are applicable both ways. The dashed line represents the line of identity.

We established conversion equations between the Q-Plex assay to reference-equivalent assay (S3 Table). We applied the conversion equations to selected biomarker values (S4 Table). For example, for a measured value of Fer by the Q-Plex assay of 13.6 μg/L there is 95% probability that the reference-equivalent value of Fer is between 13.0 μg/L and 26.7 μg/L. A graphical display of the conversion equations and 95% prediction limits are shown in Fig 1.

The median relative difference between the Q-Plex and Roche assay was: Fer -2.4%, sTfR 107%, CRP 0.03%, AGP -1.3% (Table 2). The median relative difference between the Q-Plex RBP and HPLC retinol assay was 51%. Compared to the allowable difference based on biologic variation, Fer (±7.7%), CRP (±33%), and AGP (±10%) achieved, while sTfR (±7.7%) and RBP (±12%) exceeded the minimum threshold. A different way to look at this is by calculating the percentage of Q-Plex results that agreed with the reference assay results within certain agreement limits (S5 Table). For Fer, nearly 1/3 of samples agreed within ±10%, while close to 2/3 agreed within ±30%. For CRP and AGP, nearly 50% of samples agreed within ±10% and >90% agreed within ±30%. For RBP and sTfR there were consistent assay differences; only ~30% and ~2.5% of samples agreed within ±30%, respectively.

The original sensitivity and specificity of the Q-Plex Fer (100% and 85.7%, respectively) and CRP (93.8% and 98.2%, respectively) assays was good and changed only slightly for Fer and not for CRP when we used predicted values derived from the prediction equations (Table 3). For sTfR and AGP, using predicted values led to big improvements: the original specificity for sTfR increased from 7.55% to 86.8% (sensitivity decreased); the original sensitivity for AGP increased from 44.4% to 61.1% (specificity decreased slightly). Because our sample set did not include samples with low retinol concentrations, we could not evaluate the sensitivity, but the original and predicted specificity of the Q-Plex RBP assay was 100%.

**Table 3:** Diagnostic performance of the Q-Plex assay for serum samples^a^.

	Fer		sTfR		CRP		AGP		RBP	
Parameter	Original	Predicted	Original	Predicted	Original	Predicted	Original	Predicted	Original	Predicted
Abnormal samples^b^, *n*	22 out of 78 (29%)		30 out of 83 (36%)		16 out of 72 (22%)		18 out of 85 (21%)		0 out of 85 (0%)	
Cutoff value	<15 μg/L		>5.33 mg/L		>5 mg/L		>1 g/L		<0.7 μmol/L	
True Positives, *n*	21	18	30	22	15	15	8	11	0	0
False Positives, *n*	7	1	49	7	1	1	4	8	0	0
True Negatives, *n*	50	56	4	46	55	55	63	59	85	85
False Negatives, *n*	0	3	0	8	1	1	10	7	0	0
Sensitivity^c^, %	100	85.7	100	73.3	93.8	93.8	44.4	61.1	n/a	n/a
Specificity^d^, %	87.7	98.3	7.55	86.8	98.2	98.2	94.0	88.1	100	100
PPV^e^, %	75.0	94.7	38.0	75.9	93.8	93.8	66.7	57.9	n/a	n/a
NPV^f^, %	100	94.9	100	85.2	98.2	98.2	86.3	89.4	n/a	100

^a^ AGP, α-1-acid glycoprotein; CRP, C-reactive protein; Fer, ferritin; NPV, Negative Predictive Value; PPV, Positive Predictive Value; RBP, retinol-binding protein; sTfR, soluble transferrin receptor; Roche clinical analyzer assays were used as reference assays for Fer, sTfR, CRP, and AGP; retinol measured by HPLC was used as reference assay for RBP

^b^Number of samples with concentrations below or above the cutoff value based on the reference assay

^c^Sensitivity = [True Positives / (True Positives + False Negatives)] * 100

^d^Specificity = [True Negatives / (True Negatives + False Positives)] * 100

^e^PPV = [True Positives / (True Positives + False Positives)] * 100

^f^NPV = [True Negatives / (True Negatives + False Negatives)] * 100

### Accuracy of Q-Plex based on international reference materials

The Q-Plex assay measured within ±10% of the certificate target value for Fer (-9.6%) and AGP (-1.1%), but showed larger deviations for sTfR (24%), CRP (65%), and RBP (29–37%) (Table 4). The reference assays measured mostly within ±5% of the target value except for CRP (11% lower) and sTfR (174% higher).

**Table 4:** Q-Plex and reference assay performance with international reference materials^a^.

Parameter	Fer (μg/L)	sTfR (mg/L)	CRP (mg/L)	AGP (g/L)	RBP (μmol/L)		
Reference material	NIBSC 94/572	NIBSC 07/202	ERM-DA474	ERM-DA470K	NIST SRM 968e L1	NIST SRM 968e L2	NIST SRM 968e L3
Target value^b^	6300	21.7 (60.5)^c^	41.2	0.617	1.19	1.68	2.26
Q-Plex mean (SD)^d^	5692 (145)	26.8 (3.00)	68.0 (6.22)	0.610 (0.014)	1.54 (0.064)	2.24 (0.311)	3.10 (0.311)
Q-Plex relative difference to target, %	-9.6	24 (-55)^e^	65	-1.1	29	33	37
Reference assay^f^ relative difference to target, %	5.4	174 (-1.9)^g^	-10.5	2.9	-0.7	0.5	-3.1

^a^ AGP, α-1-acid glycoprotein; CRP, C-reactive protein; ERM, European reference material; Fer, ferritin; NIBSC, National Institute for Biological Standards and Control; NIST, National Institute of Standards and Technology; RBP, retinol-binding protein; sTfR, soluble transferrin receptor

^b^ Nominal value provided in certificate

^c^ Certificate specifies target value of 21.7 mg/L (measured by absorption at 280 nm using an adjusted theoretical extinction coefficient and molecular weight calculated from the protein published sequence); Roche assay measured a value of 60.5 mg/L

^d^ Analyzed 2 replicates per material in 1 run

^e^ Quansys assay measured 24% higher than the certificate value and 55% lower compared to the Roche determined target value

^f^ Roche clinical analyzer assays were used as reference assays for Fer, sTfR, CRP, and AGP; retinol measured by HPLC was used as reference assay for RBP

^g^ Roche assay measured 174% higher than the certificate value and 1.9% lower compared to the original material characterization in 2008

### Imprecision of Q-Plex

The mean intra-assay imprecision showed CVs of <5%: Fer 2.1%, sTfR 4.5%, CRP 2.3%, AGP 3.2%, and RBP 2.3% (Table 5). The mean inter-assay CVs were higher: Fer 11%, sTfR 14%, CRP 9.3%, AGP 7.5%, and RBP 19%. When we compared the inter-assay CVs to the allowable imprecision based on biologic variation, CRP (32%) and AGP (8.5%) achieved, Fer (11%) borderline achieved, and sTfR (8.5%) and RBP (7.8%) exceeded the minimum threshold. In contrast, all 5 reference assays achieved the minimum imprecision threshold and in most cases achieved the optimum threshold (S2 Table).

**Table 5:** Q-Plex and reference assay imprecision for serum samples^a^.

	Fer		sTfR		CRP		AGP		RBP	
Parameter	Concentration (μg/L)	CV (%)	Concentration (mg/L)	CV (%)	Concentration (mg/L)	CV (%)	Concentration (g/L)	CV (%)	Concentration (μmol/L)	CV (%)
Q-Plex intra-assay^b^										
Sample 1	14.3	3.5	9.42	4.6	2.18	1.4	0.844	2.7	3.84	2.4
Sample 2	13.4	2.3	21.0	4.2	5.73	1.9	0.960	3.6	1.15	2.3
Sample 3	112	1.4	15.6	5.3	0.745	2.7	0.696	2.4	2.24	1.9
Sample 4	586	1.3	10.2	3.8	0.896	3.1	1.11	4.3	3.32	2.5
*Mean CV*, *%*		*2*.*1*		*4*.*5*		*2*.*3*		*3*.*2*		*2*.*3*
Q-Plex inter-assay^c^										
Sample 1	12.2	14	7.49	17	2.10	8.3	0.794	6.7	2.49	28
Sample 2	13.9	9.2	19.2	15	7.16	10	0.993	7.6	1.11	11
Sample 3	113	6.5	13.9	14	0.821	6.7	0.665	8.8	1.99	15
Sample 4	512	13	9.05	9.8	0.905	12	1.08	6.9	2.60	22
*Mean CV*, *%*		*11*		*14*		*9*.*3*		*7*.*5*		*19*
Reference inter-assay^d^										
Low QC	11.5	1.7	2.92	1.5	1.26	2.7	0.792	2.9	0.761	6.1
High QC	70.8	1.8	12.6	1.8	23.0	1.9	1.52	1.9	1.57	6.0
Minimum allowable analytical CV^e^		11		8.5		32		8.5		7.8

^a^ AGP, α-1-acid glycoprotein; CRP, C-reactive protein; Fer, ferritin; RBP, retinol-binding protein; sTfR, soluble transferrin receptor

^b^ Q-Plex intra-assay imprecision was assessed by analyzing 5 replicates per sample in 1 experiment

^c^ Q-Plex inter-assay imprecision was assessed by analyzing a single replicate per sample in 10 experiments

^d^ Reference assay inter-assay imprecision was assessed by analyzing duplicates per sample in 10 experiments; Roche clinical analyzer assays were used as reference assays for Fer, sTfR, CRP, and AGP; retinol measured by HPLC was used as reference assay for RBP

^e^ Biologic variation was used to derive objective quality goal for method imprecision (see S2 Table); allowable minimum imprecision = 0.75*within-individual biologic variation

### Dilution linearity of Q-Plex

The Quansys kit instructions indicate to dilute samples at least 1:10. We evaluated whether lower or higher dilution can be used to measure samples with concentrations outside the reportable range. The mean recovery varied by analyte and dilution (S2 Fig). Assuming an acceptable recovery tolerance of 85–115%, Fer (115%), sTfR (85%), and AGP (103%) showed acceptable, while CRP (165%) and RBP (173%) showed unacceptable recoveries at 1:5 dilution. A 1:20 dilution was acceptable for Fer (86%), CRP (92%), and AGP (105%), but unacceptable for sTfR (118%) and RBP (79%). A 1:40 dilution showed acceptable recovery for CRP (95%) and AGP (105%), but unacceptable recoveries for Fer (84%), sTfR (135%), and RBP (65%).

### Effect of specimen matrices

While some matrix differences were statistically significant (*P* <0.05), they were not biologically relevant and all were within ±5% of the serum results (Table 6). However, the Q-Plex sTfR results for EDTA-P were 74% higher than serum results. The Roche sTfR assay did not show such an effect.

**Table 6:** Q-Plex and reference assay results for matrix-matched samples^a^.

Assay and sample matrix	Fer (μg/L)	sTfR (mg/L)	CRP (mg/L)	AGP (g/L)	RBP (μmol/L)
Q-Plex assay					
Serum	45.3 (26.5, 64.1)	13.2 (9.22, 17.3)	2.66 (1.80, 3.52)	0.829 (0.751, 0.906)	2.86 (2.49, 3.24)
Heparin plasma	42.9 (25.5, 60.3)	13.0 (9.19, 16.7)	2.69 (1.77, 3.61)	0.789 (0.713, 0.865)	2.79 (2.37, 3.22)
EDTA plasma	43.8 (24.7, 62.9)	23.7 (15.2, 32.2)	2.72 (1.72, 3.73)	0.836 (0.751, 0.921)	3.02 (2.57, 3.47)
*P* value (heparin plasma)	0.0204	0.13	0.99	0.0003	0.48
*P* value (EDTA plasma)	0.06	0.0002	0.67	0.53	0.15
Reference assay^b^					
Serum	39.5 (25.5, 53.4)	5.68 (4.33, 7.04)	2.60 (1.79, 3.40)	0.805 (0.714, 0.895)	1.61 (1.46, 1.76)
Heparin plasma	35.5 (24.8, 51.6)	5.54 (4.20, 6.87)	2.54 (1.73, 3.33)	0.778 (0.691, 0.865)	1.55 (1.41, 1.69)
EDTA plasma	38.2 (24.7, 51.6)	6.05 (4.66, 7.43)	2.41 (1.63, 3.18)	0.768 (0.681, 0.856)	1.55 (1.39, 1.70)
*P* value (heparin plasma)	0.0005	<0.0001	0.0099	<0.0001	0.0078
*P* value (EDTA plasma)	0.0002	<0.0001	0.12	<0.0001	0.06

^a^ AGP, α-1-acid glycoprotein; CRP, C-reactive protein; Fer, ferritin; RBP, retinol-binding protein; sTfR, soluble transferrin receptor; arithmetic mean (95% CI) of 25 paired serum, heparin plasma, and EDTA plasma samples; 2 samples for Fer, 1 sample for sTfR, and 4 samples for CRP were excluded because they were out of calibrator range for the Q-Plex assay

^b^ Roche clinical analyzer assays were used as reference assays for Fer, sTfR, CRP, and AGP; retinol measured by HPLC was used as reference assay for RBP

### Freeze-thaw stability of Q-Plex

For serum samples, we observed for all analytes acceptable differences of ≤±10% compared to the reference condition for up to 5 freeze-thaw cycles, with AGP showing a small but consistent positive difference (S6 Table). For HEP-P samples, we observed slightly larger differences of ≤±17% compared to the reference condition, with Fer and sTfR showing consistent positive differences and CRP, AGP, and RBP showing consistent negative differences. For EDTA-P samples, nearly all results were within ±10% compared to the reference condition, but we observed consistent negative differences for sTfR, CRP, and RBP. The sTfR results in EDTA-P were again much higher than in serum (62%), but they did not increase with additional freeze-thaw cycles.

### Effect of incubation temperature

When we subjected serum, HEP-P, and EDTA-P samples to an elevated incubation temperature of 30°C compared to our room temperature of 18°C (suitable per manufacturer), the Q-Plex assay produced similar results for CRP (5.8% higher), slightly lower results for Fer (-8.6%), much lower results for AGP (-39%) and RBP (all results were <LOD and could not be calculated), and much higher results for sTfR (88%) (S7 Table). Moreover, the calibration curves at 30°C displayed different shapes and the background noise was increased, resulting in lower assay sensitivity. As such, we had to exclude 3 samples with low Fer and 2 samples with low CRP concentrations from this experiment, because results could not be calculated after the 30°C incubation.

## Discussion

This study is to our knowledge the first to compare the Quansys 5-plex microarray with well-established and validated reference-type assays. Furthermore, we carefully conducted the statistical analysis to assess the agreement between the test and reference assay, ensuring that we appropriately address assumption violations such as non-constant variance and non-constant difference. Because previous method comparison studies [8,11] used different approaches, it is difficult to compare the findings.

As such, the median relative differences between the Q-Plex and reference assay in our study (Fer -2.4%, sTfR 107%, CRP 0.03%, AGP -1.3%, and RBP 51%) do not correspond well with the 2 previously reported relative differences between the Q-Plex and the commonly-used sandwich ELISA [8,11]: Fer 88% and 108%, sTfR 70% and 148%, CRP -33% and -1%, AGP -53% and -37%, and RBP -16% and 12%. Several reasons could explain these discrepancies: different antibodies with different specificities and affinities used in these comparisons; different sample sets may result in different assay relationships either due to different concentration ranges and/or due to sample composition; the statistical approach used to assess the assay agreement varies across studies and non-constant variance and/or non-constant differences may not have been addressed in previous studies; in previous studies, the authors used the slope to describe the proportional difference between the assays without giving consideration to the intercept, which in some cases was quite large. It would be interesting to see how our findings compare to the previous studies if those studies used the same statistical approach.

The prediction equations derived in this study allow the conversion of Q-Plex data to reference assay-equivalent data, but they should be considered preliminary until further confirmation. Conversion equations may be helpful in the future to allow comparison of Q-Plex assay data with US population data generated with the reference-type assays in the National Health and Nutrition Examination Survey. Previous studies that compared the Q-Plex and sandwich-ELISA assay with samples from African and Asian countries, appeared to cover similar concentration ranges to our study based on visual inspection of the scatter plots or Bland-Altman plots [8,11]. Still, the equations derived in this study should be confirmed with other sample sets, particularly from countries where analyte concentrations are different from those in the United States.

Assessing the accuracy of an assay with international reference materials is difficult due to potential commutability issues, i.e., the assay responds differently to the reference material compared to a native sample. The Roche CRP assay measured 11% lower than the certificate target value, due to the non-commutability of the ERM reference material, possibly because it is a spiked material. However, Roche has confirmed that the CRP (Gen.3) assay measures accurately for patient samples (personal communication with Guenter Trefz, Roche, 06/08/2016). Similarly, the Q-Plex CRP assay measured closely to the Roche assay (0.03% difference) in serum samples, but 65% higher in the ERM reference material, suggesting non-commutability of the reference material for the Q-Plex assay. Furthermore, the Q-Plex sTfR assay measured 107% higher than the Roche assay in serum samples, but 55% lower in the NIBSC reference material, possibly indicating non-commutability of the reference material for the Q-Plex assay. The Q-Plex RBP assay measured 51% higher than the HPLC retinol assay in serum samples and 29–37% higher in the NIST reference material, a difference of similar magnitude, suggesting that the reference material is likely commutable for the Q-Plex assay. Another difficulty in interpreting data from reference materials is the lack of assay standardization. The Roche sTfR assay has not been calibrated to the NIBSC reference material and measures much higher (174%) than the certificate target value [15]. However, the assay has largely remained stable over the last 10 years, still producing the same value as during the original material characterization in 2008.

The low intra-assay imprecision in this study was similar to a previous report [8]. However, the inter-assay imprecision in this study was mostly higher compared to a previous report [8]: Fer 11% *vs*. 6.2–8.7%, sTfR 14% *vs*. 9.9–13.9%, CRP 9.3% *vs*. 6.4–8.7%, AGP 7.5% *vs*. 5.3–9.6%, and RBP 19% *vs*. 10.0–12.3%.

The performance of the Q-Plex CRP assay was best among the 5 analytes, meeting the performance criteria for precision and difference to the reference assay, as well as showing high (>90%) sensitivity and specificity before and after adjusting the data with the prediction equation.

The Q-Plex Fer assay performed second best, meeting the performance criteria for precision (borderline) and difference to the reference assay, as well as both showing high (>85%) sensitivity and specificity before and after adjusting the data with the prediction equation. The relationship between the 2 assays was most complicated for Fer because it was non-linear and showed increasing variance. Even though the Q-Plex assay measured lower at low Fer and higher at high Fer concentrations compared to the Roche assay, the diagnostic performance of the assay was still satisfactory.

The Q-Plex AGP assay met the performance criteria for precision and difference to the reference assay, showed high specificity (>85% before and after adjustment), but showed only moderate sensitivity (44.4% before and 61.1% after adjustment).

The Q-Plex sTfR assay did not meet the performance criteria for precision and difference to the reference assay and showed poor specificity (7.55%) due to an apparent calibration difference; after adjusting the data with the prediction equation, the specificity improved (>85%) at the cost of a loss in sensitivity (100% to 73.3%). An improvement in sTfR precision would be desirable.

Lastly, the Q-Plex RBP assay did not meet the performance criteria for precision and difference to the reference assay, and we could not assess the sensitivity of the assay because our sample set did not contain samples with low retinol concentrations. Given that the difference to the reference assay was of similar magnitude for serum samples and the NIST reference material, it may be possible to adjust the Q-Plex assay. However, an improvement in RBP precision would still be necessary. Because our comparison of RBP to retinol is a combined biomarker validity/analytical comparability assessment, it is hard to separate between the methodological and the potential physiologic components. However, our ultimate goal was to assess how the RBP Q-Plex assay compared to serum retinol, which is currently the generally accepted biomarker for vitamin A deficiency.

While the method comparison information is the center-piece of this paper, we also tested relevant pre-analytical factors that users need to be aware of. We confirmed that serum samples cannot be diluted less than 1:10 and that a 1:20 dilution leads to >±15% deviation for sTfR and RBP. We showed that EDTA-P is not a suitable sample matrix for the Q-Plex assay because of invalid high sTfR results. A similar effect has been observed previously with other ELISA sTfR assays [16] and is possibly due to an interference of EDTA with the antibody. If serum cannot be obtained, HEP-P is a suitable alternative. All 5 analytes showed good stability for ≤5 freeze-thaw cycles, with serum showing less difference from the reference condition than the 2 plasma matrices. The Q-Plex assays did not show any notable interference from repeated freeze-thawing. Lastly, we showed that elevated temperature (30°C) during the performance of the Q-Plex assay is problematic, as it leads to invalid high sTfR, low AGP, and non-detectable RBP results.

In conclusion, the Quansys 5-plex microarray has a number of advantages compared to conventional laboratory assays that makes it attractive for low-resource settings: it is easy to perform, requires minimal analyst training, needs only a small sample volume, is relatively inexpensive, and measures all 5 biomarkers at the same time (Table 1). However, some improvements in accuracy and precision are still desirable and the inclusion of quality control materials in the kit is needed so that the user can monitor consistency and assay performance.

## Supporting information

S1 Fig Difference plots showing the difference between the test and the reference assay on the y-axis and the average of the 2 assays on the x-axis. Panels A-E show the original data and panels F-J show the log-transformed data; ferritin (panels A and F), soluble transferrin receptor (panels B and G), C-reactive protein (panels C and H), α-1-acid glycoprotein (panels D and I), and retinol-binding protein (panels E and J). The solid horizontal line represents the zero-line. The dashed horizontal lines represent the 2.5^th^ and 97.5^th^ percentiles for the data shown. The dashed linear regression line is used to assess non-constant difference.
(TIF)

S2 Fig Dilution recovery for 5 serum samples diluted at lower or higher sample dilution relative to the recommended dilution (1:10). Error bars represent the 95% CI. Dashed lines represent 100% recovery ± 15% tolerance limits. AGP, α-1-acid glycoprotein; CRP, C-reactive protein; dil, dilution; Fer, ferritin; RBP, retinol-binding protein; sTfR, soluble transferrin receptor.
(TIF)

S1 Text Detailed information on analytical methods. (DOCX)

S2 Text Detailed information on experiments assessing the effect of pre-analytical factors on Q-Plex results. (DOCX)

S3 Text Detailed information on statistical analysis. (DOCX)

S1 Table Q-Plex calibration range. Ranges are calibrator lot specific (shown for lot HMTM170411); concentrations shown represent raw concentrations in calibration curve; samples are diluted 1:10.
(DOCX)

S2 Table Objective quality goals for method performance based on biologic variation. References for the objective quality goals for each analyte are provided in the first 2 rows showing the within- and between-individual variation; AGP, α-1-acid glycoprotein; CRP, C-reactive protein; CV_A_, analytical variation; CV_G_, between-individual or group variation; CV_I_, within-individual variation; D, difference to target; Fer, ferritin; RBP, retinol-binding protein; Ref, reference; sTfR, soluble transferrin receptor.
(DOCX)

S3 Table Selected conversion equations between the Q-Plex and reference assay for serum samples. AGP, α-1-acid glycoprotein; CRP, C-reactive protein; Fer, ferritin; RBP, retinol-binding protein; sTfR, soluble transferrin receptor; Roche clinical analyzer assays used as reference assays for Fer, sTfR, CRP, and AGP; retinol measured by HPLC used as reference assay for RBP; prediction error (PE) is provided in parentheses and can be used to construct 95% prediction intervals for a selected value x using ± t_n-1,0.025_PE, where t_*n*-1,0.025_ is the 97.5^th^ percentile from the Student *t* distribution with *n*-1 degrees of freedom.
(DOCX)

S4 Table Predictions and 95% prediction intervals from conversion equations. Results in this table can be interpreted as follows: for a future measured value by assay x there is a 95% probability that the future measured value by assay y would be contained in the presented prediction interval. AGP, α-1-acid glycoprotein; CRP, C-reactive protein; Fer, ferritin; RBP, retinol-binding protein; sTfR, soluble transferrin receptor; Roche clinical analyzer assays used as reference assays for Fer, sTfR, CRP, and AGP; retinol measured by HPLC used as reference assay for RBP.
(DOCX)

S5 Table Percentage of Q-Plex serum sample results that agree with the reference assay results within selected limits. AGP, α-1-acid glycoprotein; CRP, C-reactive protein; Fer, ferritin; RBP, retinol-binding protein; sTfR, soluble transferrin receptor; Roche clinical analyzer assays were used as reference assays for Fer, sTfR, CRP, and AGP; retinol measured by HPLC was used as reference assay for RBP.
(DOCX)

S6 Table Q-Plex freeze-thaw stability of serum, heparin plasma, and EDTA plasma samples. AGP, α-1-acid glycoprotein; CRP, C-reactive protein; Fer, ferritin; RBP, retinol-binding protein; sTfR, soluble transferrin receptor; 5 serum, heparin plasma, and EDTA plasma samples subjected to up to 5 freeze-thaw cycles (3 h at room temperature/cycle), then analyzed together with the reference samples (no freeze-thaw cycle) in the same run; samples stored at -70°C when not in use. Mean concentration across 5 samples; the SD estimates the variability at the reference condition for each matrix. Percent difference to reference condition was calculated for each sample and then averaged across 5 samples.
(DOCX)

S7 Table Q-Plex results comparing incubation at 30°C with incubation at 18°C. AGP, α-1-acid glycoprotein; CRP, C-reactive protein; Fer, ferritin; RBP, retinol-binding protein; sTfR, soluble transferrin receptor. Mean concentration across ~35–40 samples consisting of about 1/3 serum, 1/3 heparin plasma, and 1/3 EDTA plasma samples; the SD estimates the variability at the 2 incubation temperatures. Percent difference to incubation temperature of 18°C was calculated for each sample and then averaged across all samples. Analysis is based on same number of samples analyzed at both incubation temperatures.
(DOCX)

S1 Data Comparison with reference assays. (XLSX)

S2 Data Reference materials_Table 4. (XLSX)

S3 Data Imprecision_Table 5. (XLSX)

S4 Data Dilution linearity_S2 Fig. (XLSX)

S5 Data Matrix_Table 6. (XLSX)

S6 Data Freeze-Thaw Stability_S6 Table. (XLSX)

S7 Data Incubation temperature_S7 Table. (XLSX)
