# Supplementary figures and images for: The Quansys multiplex immunoassay for serum ferritin, C-reactive protein, and α-1-acid glycoprotein showed good comparability with reference-type assays but not for soluble transferrin receptor and retinol-binding protein
Source: PLoS One. 2019 Apr 29;14(4):e0215782. doi: 10.1371/journal.pone.0215782 (PMC6488062; doi:10.1371/journal.pone.0215782)

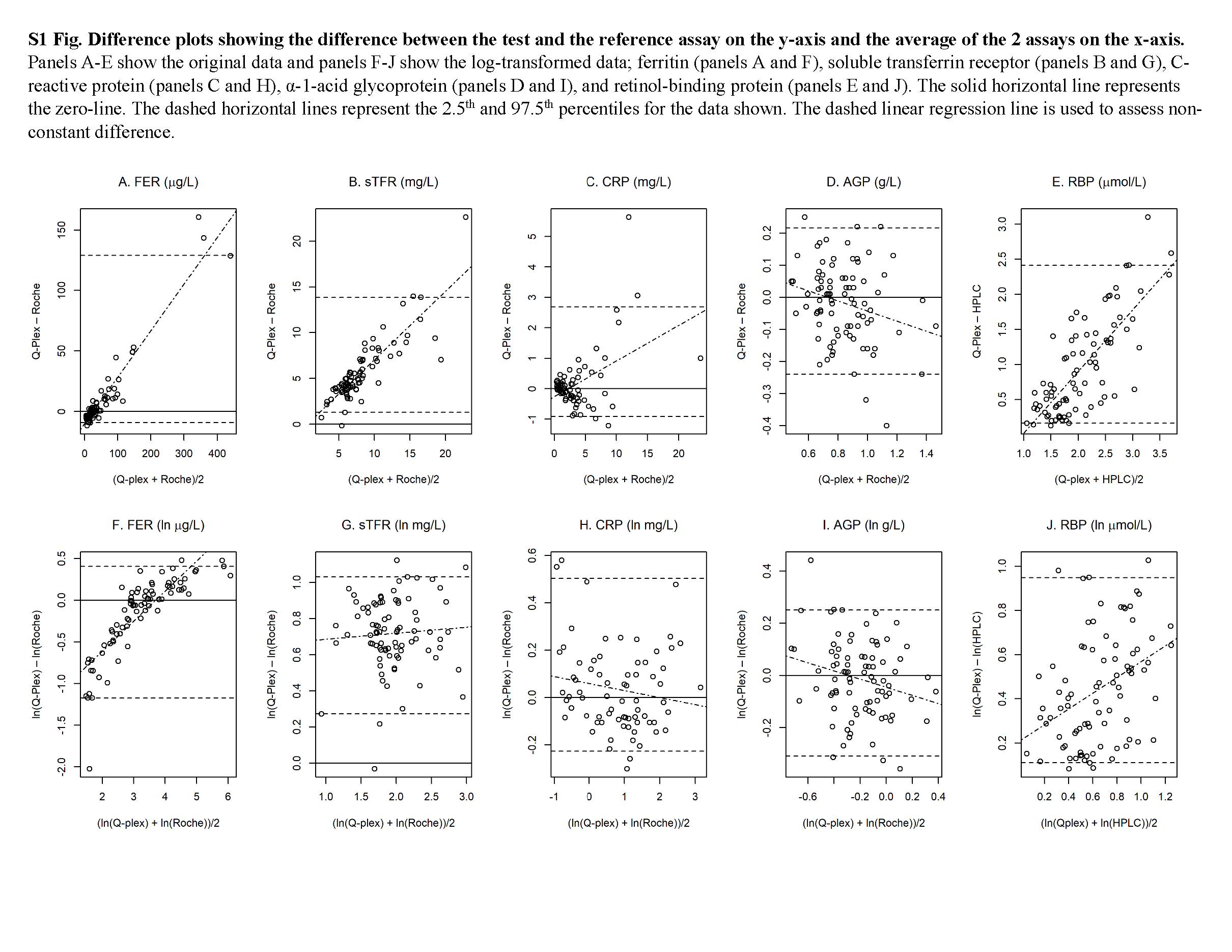

Supplement: S1 Fig — Panels A-E show the original data and panels F-J show the log-transformed data; ferritin (panels A and F), soluble transferrin receptor (panels B and G), C-reactive protein (panels C and H), α-1-acid glycoprotein (panels D and I), and retinol-binding protein (panels E and J). The solid horizontal line represents the zero-line. The dashed horizontal lines represent the 2.5th and 97.5th percentiles for the data shown. The dashed linear regression line is used to assess non-constant difference. (TIF) [file pone.0215782.s001.tif]

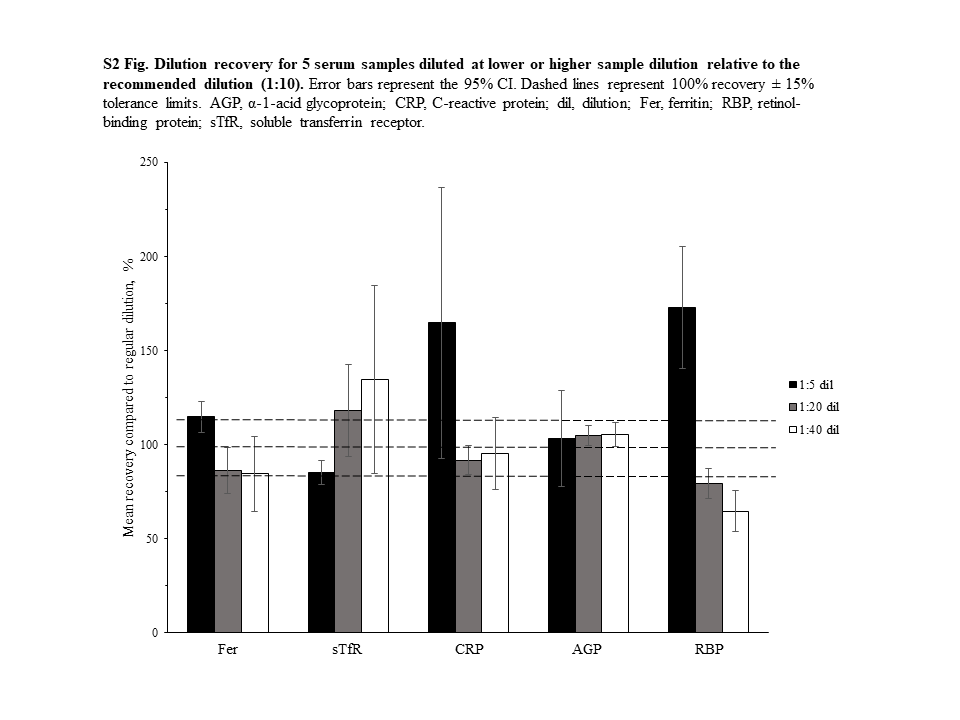

Supplement: S2 Fig — Error bars represent the 95% CI. Dashed lines represent 100% recovery ± 15% tolerance limits. AGP, α-1-acid glycoprotein; CRP, C-reactive protein; dil, dilution; Fer, ferritin; RBP, retinol-binding protein; sTfR, soluble transferrin receptor. (TIF) [file pone.0215782.s002.tif]
