# Supplementary material for: The Quansys multiplex immunoassay for serum ferritin, C-reactive protein, and α-1-acid glycoprotein showed good comparability with reference-type assays but not for soluble transferrin receptor and retinol-binding protein
Source: PLoS One. 2019 Apr 29;14(4):e0215782. doi: 10.1371/journal.pone.0215782 (PMC6488062; doi:10.1371/journal.pone.0215782)
Supplement: S1 Text — (DOCX) [file pone.0215782.s003.docx]

**S1 Text.** **Detailed information on analytical methods**

*Quansys multiplex*

The Quansys multiplex assay (Q-Plex™) kit contains a 96-well plate coated with capture antibody featuring the 5-plex and a reference spot in each well, and all reagents required to carry out the assay (calibrator, competitor, sample diluent, wash buffer, detection mix, streptavidin-HRP, and substrates). We used the Q-View Imager LS and Q-View software from Quansys Biosciences to collect the results in each experiment. Our study used 13 5-plex kits with the same calibrator lot HMTM170411, and the software product code was HMTM170411CD-C17. For the accuracy testing using reference materials, we used a 7-plex plate with calibrator lot HMTM170411-C18 and software product code HMTM170411MN-C18.

The assay principle uses a sandwich ELISA for Fer and sTfR, and a competitive immunoassay for AGP, CRP, and RBP. The Q-Plex™ kit uses lyophilized recombinant antigens in a buffered protein base as calibrators. We strictly followed the kit instructions to prepare reagents and perform all experiments. We reconstituted the lyophilized calibrator in Quansys diluent and further serially diluted it to yield 7-point calibration curves. According to the kit instructions, 1 out of 7 points will be automatically excluded from the calibration curve if the calculated concentration is >±20% outside the target value, or if the imprecision (CV) between the replicates is >30%.

*Reference assays for Fer, sTfR, CRP, and AGP analysis by Roche clinical analyzer*

The Roche cobas 6000 clinical analyzer was used as a reference method to compare Fer, sTfR, CRP, and AGP concentrations. The e601 immunoanalyzer was used for Fer (electrochemiluminescence immunoassay) and the c501 chemistry analyzer for sTfR (Tina-quant; particle enhanced immunoturbidimetric assay), CRP (Gen.3; particle enhanced immunoturbidimetric assay), and AGP (Tina-quant Gen.2; immunoturbidimetric assay). The reportable range for each analyte was as follows: Fer 0.5–2000 µg/L, sTfR 0.5–40 mg/L*,* CRP 0.3–350 mg/L, and AGP 0.1–4.0 g/L.

*Reference assay for vitamin A by HPLC-UV analysis*

Vitamin A (retinol) concentrations were measured using an in-house high performance liquid chromatography (HPLC) assay with UV detection. Twenty-five µL of serum was mixed with an internal standard (retinyl acetate). After liquid-liquid extraction, the supernatant was injected into the HPLC. Chromatograms were recorded at a wavelength of 325 nm. Quantitation was performed using peak height ratio of the analyte to internal standard. The reportable range was 0.03–5.24 µmol/L.
