# Supplementary material for: The Quansys multiplex immunoassay for serum ferritin, C-reactive protein, and α-1-acid glycoprotein showed good comparability with reference-type assays but not for soluble transferrin receptor and retinol-binding protein
Source: PLoS One. 2019 Apr 29;14(4):e0215782. doi: 10.1371/journal.pone.0215782 (PMC6488062; doi:10.1371/journal.pone.0215782)
Supplement: S2 Text — (DOCX) [file pone.0215782.s004.docx]

**S2 Text. Detailed information on experiments assessing the effect of pre-analytical factors on Q-Plex^TM^ results**

***Dilution linearity of Q-Plex™***

Five serum samples with different biomarker concentrations were diluted at lower (1:5) and higher (1:20 and 1:40) sample dilution compared to the recommended 1:10 dilution. We calculated the dilution recovery in percent relative to the 1:10 dilution results.

***Matrix-matched samples analyzed by Q-Plex™ and reference assays***

We assessed the Q-Plex™ performance using 25 matrix-matched pairs of serum, HEP-P, and EDTA-P. Paired samples were analyzed on the same plate to minimize analytical variability. We also measured the same samples with the reference assays (paired samples in the same run).

***Freeze-thaw stability of Q-Plex™***

We investigated the analytes’ freeze-thaw stability with the Quansys assay using 5 samples and the 3 available matrices per sample (serum, HEP-P, and EDTA-P). Each sample was aliquoted into 6 vials and stored at -70°C overnight. One vial for each sample (reference; no additional freeze-thaw cycles) was kept frozen until batch analysis at the conclusion of the experiment. The other vials were subjected to up to 5 freeze-thaw cycles (3 h at room temperature per cycle). All 6 vials for each sample were analyzed on 1 plate to minimize analytical variation.

***Effect of incubation temperature on Q-Plex™ results***

The Quansys kit instructions state that plates should be incubated at room temperature (20–25°C). Quansys technical support confirmed that a lower room temperature of 18°C (in our laboratory) will not cause any issues with the assay. However, we investigated the effect of plate incubation at higher than recommended temperature to mimic conditions in some low-resource laboratories. We used a controlled temperature incubator set to 30°C and placed a plate shaker inside the incubator. Approximately 35–40 samples, made up of about 1/3 serum, 1/3 EDTA-P, and 1/3 HEP-P samples were diluted 1:10 and dispensed into 2 plates. One plate (reference) was incubated at room temperature (18°C), while the other plate was shaken in the incubator at 30°C throughout the entire experiment (140 min).
