# Supplementary material for: The Quansys multiplex immunoassay for serum ferritin, C-reactive protein, and α-1-acid glycoprotein showed good comparability with reference-type assays but not for soluble transferrin receptor and retinol-binding protein
Source: PLoS One. 2019 Apr 29;14(4):e0215782. doi: 10.1371/journal.pone.0215782 (PMC6488062; doi:10.1371/journal.pone.0215782)
Supplement: S3 Text — (DOCX) [file pone.0215782.s005.docx]

**S3 Text.** **Detailed information on statistical analysis**

Bland and Altman’s limits of agreement (LoA) method is commonly used to assess the agreement between 2 assays. However, the LoA approach is not appropriate when the variance of the difference between the assays is not constant over the range of measurements, the difference between the 2 assays is not constant, or the variances of the measurement errors are different for each assay. A variation of the LoA method has been suggested [1, 2], using standard linear regression of the difference between the assays on the average of the assays to derive prediction equations from one assay (test) to the other (reference). In this way, there is a 1:1 correspondence between prediction intervals and the LoA obtained with the Bland-Altman approach [1]. The prediction interval approach can accommodate non-linear relationships or non-constant variance by transforming the data. A 95% prediction interval provides limits for which 95% of future measurements from one method, given a specified measurement by another method are expected to fall.

While one can assess departures from constant variance and constant difference in a Bland-Altman plot (difference plot) by eye, we supplemented this approach with statistical tests. A hypothesis test on the slope coefficient (H_0_: β=0) from a regression of the differences on the averages provides a test of a non-constant difference. Using the absolute residuals from this regression, a hypothesis test on the slope coefficient from a regression of these absolute residuals on the averages provides a test for non-constant variance.

**References**

[1] Carstensen B. Comparing clinical measurement methods: a practical guide, John Wiley & Sons, 2011.

[2] Bland JM, Altman DG Measuring agreement in method comparison studies, Statistical methods in medical research 8, 1999, pp.135–160.
