# Supplementary material for: The Quansys multiplex immunoassay for serum ferritin, C-reactive protein, and α-1-acid glycoprotein showed good comparability with reference-type assays but not for soluble transferrin receptor and retinol-binding protein
Source: PLoS One. 2019 Apr 29;14(4):e0215782. doi: 10.1371/journal.pone.0215782 (PMC6488062; doi:10.1371/journal.pone.0215782)
Supplement: S1 Table — Ranges are calibrator lot specific (shown for lot HMTM170411); concentrations shown represent raw concentrations in calibration curve; samples are diluted 1:10. (DOCX) [file pone.0215782.s006.docx]

**S1 Table.** **Q-Plex™ calibration range^a^**

| **Parameter** | **Fer (µg/L)** | **sTfR (mg/L)** | **CRP (mg/L)** | **AGP (g/L)** | **RBP (µmol/L)** |
| --- | --- | --- | --- | --- | --- |
| Calibrator concentration | 114 | 121 | 5.19 | 0.37 | 1.01 |
| Upper Limit of Quantitation | 109.7 | 121 | 5.19 | 0.36 | 0.98 |
| Lower Limit of Quantitation | 0.17 | 0.17 | 0.011 | 0.0015 | 0.0042 |
| Limit of Detection | 0.18 | 0.13 | 0.0094 | 0.00061 | 0.0012 |

^a^ Ranges are calibrator lot specific (shown for lot HMTM170411); concentrations shown represent raw concentrations in calibration curve; samples are diluted 1:10
