# Supplementary material for: The Quansys multiplex immunoassay for serum ferritin, C-reactive protein, and α-1-acid glycoprotein showed good comparability with reference-type assays but not for soluble transferrin receptor and retinol-binding protein
Source: PLoS One. 2019 Apr 29;14(4):e0215782. doi: 10.1371/journal.pone.0215782 (PMC6488062; doi:10.1371/journal.pone.0215782)
Supplement: S2 Table — References for the objective quality goals for each analyte are provided in the first 2 rows showing the within- and between-individual variation; AGP, α-1-acid glycoprotein; CRP, C-reactive protein; CVA, analytical variation; CVG, between-individual or group variation; CVI, within-individual variation; D, difference to target; Fer, ferritin; RBP, retinol-binding protein; Ref, reference; sTfR, soluble transferrin receptor. (DOCX) [file pone.0215782.s007.docx]

**S2 Table.** **Objective quality goals for method performance based on biologic variation [1]^a^**

| **Parameter** | **Fer** | **sTfR** | **CRP** | **AGP** | **RBP** |
| --- | --- | --- | --- | --- | --- |
| Within-individual CV_I_, % [Ref] | 14.2 [2] | 11.3 [3] | 42.2 [2] | 11.3 [2] | 10.4 [5] |
| Between-individual CV_G_, % [Ref] | 15.0 [2] | 17.3 [4] | 76.3 [2] | 24.9 [2] | 37.9 [5] |
| Allowable imprecision, % |  |  |  |  |  |
| Optimum, CV_A_=0.25*CV_I_ | 3.6 | 2.8 | 10.6 | 2.8 | 2.6 |
| Desirable, CV_A_=0.5*CV_I_ | 7.1 | 5.7 | 21.1 | 5.7 | 5.2 |
| Minimum, CV_A_=0.75*CV_I_ | 10.7 | 8.5 | 31.7 | 8.5 | 7.8 |
| Allowable difference to target, % |  |  |  |  |  |
| Optimum, D=0.125*(CV_I_^2^+CV_G_^2^)^1/2^ | 2.6 | 2.6 | 10.9 | 3.4 | 4.1 |
| Desirable, D=0.25*(CV_I_^2^+CV_G_^2^)^1/2^ | 5.2 | 5.2 | 21.8 | 6.8 | 8.2 |
| Minimum, D=0.375*(CV_I_^2^+CV_G_^2^)^1/2^ | 7.7 | 7.7 | 32.7 | 10.3 | 12.3 |

^a^ References for the objective quality goals for each analyte are provided in the first 2 rows showing the within- and between-individual variation; AGP, α-1-acid glycoprotein; CRP, C-reactive protein; CV_A_, analytical variation; CV_G_, between-individual or group variation; CV_I_, within-individual variation; D, difference to target; Fer, ferritin; RBP, retinol-binding protein; Ref, reference; sTfR, soluble transferrin receptor

**References**

[1] Fraser CG, Petersen PH, Libeer JC, Ricos *C.* Proposals for setting generally applicable quality goals solely based on biology. *Annals of clinical biochemistry*.1997; 34, 8-12.

[2] Ricós C, Alvarez V, Cava F, Garcia-Lario J, Hernandez A, Jimenez C, *et al.* Desirable specifications for total error, imprecision, and bias, derived from intra-and inter-individual biologic variation. Update. 2014; 1539-45.

[3] Cooper MJ, Zlotkin SH*.* Day-to-day variation of transferrin receptor and ferritin in healthy men and women, The American journal of clinical nutrition. 1996; 64, 738-742.

[4] Raya G, Henny J., Steinmetz J, Herbeth B, Siest G*.* Soluble transferrin receptor (sTfR): biological variations and reference limits, Clinical chemistry and laboratory medicine.2001; 39, 1162-1168.

[5] Lacher DA, Hughes JP, Carroll MD*.* Biological variation of laboratory analytes based on the 1999-2002 National Health and Nutrition Examination Survey. National Health Statistics Reports; no 21. Hyattsville, MD: National Center for Health Statistics. 2010.
