# Supplementary material for: The Quansys multiplex immunoassay for serum ferritin, C-reactive protein, and α-1-acid glycoprotein showed good comparability with reference-type assays but not for soluble transferrin receptor and retinol-binding protein
Source: PLoS One. 2019 Apr 29;14(4):e0215782. doi: 10.1371/journal.pone.0215782 (PMC6488062; doi:10.1371/journal.pone.0215782)
Supplement: S3 Table — AGP, α-1-acid glycoprotein; CRP, C-reactive protein; Fer, ferritin; RBP, retinol-binding protein; sTfR, soluble transferrin receptor; Roche clinical analyzer assays used as reference assays for Fer, sTfR, CRP, and AGP; retinol measured by HPLC used as reference assay for RBP; prediction error (PE) is provided in parentheses and can be used to construct 95% prediction intervals for a selected value x using ± tn-1,0.025PE, where tn-1,0.025 is the 97.5th percentile from the Student t distribution with n-1 degrees of freedom. (DOCX) [file pone.0215782.s008.docx]

**S3 Table.** **Selected conversion equations between the Q-Plex™ and reference assay for serum samples^a^**

| **Parameter** | **Fer (µg/L)** | **sTfR (mg/L)** | **CRP (mg/L)** | **AGP (g/L)** | **RBP (µmol/L)** |
| --- | --- | --- | --- | --- | --- |
| Q-Plex™ is x; reference is y | ln y=1.10+0.70*ln x (0.18) | ln y=–0.72+ln x (0.20) | ln y=–0.03+ln x (0.18) | y=–0.12+1.17x (0.13) | ln y=–0.18+0.70*ln x (0.20) |

^a^ AGP, α-1-acid glycoprotein; CRP, C-reactive protein; Fer, ferritin; RBP, retinol-binding protein; sTfR, soluble transferrin receptor; Roche clinical analyzer assays used as reference assays for Fer, sTfR, CRP, and AGP; retinol measured by HPLC used as reference assay for RBP; prediction error (PE) is provided in parentheses and can be used to construct 95% prediction intervals for a selected value x using ± t_n-1,0.025_PE, where t*_n_*_-1,0.025_ is the 97.5^th^ percentile from the Student *t* distribution with *n*-1 degrees of freedom
