# Supplementary material for: The Quansys multiplex immunoassay for serum ferritin, C-reactive protein, and α-1-acid glycoprotein showed good comparability with reference-type assays but not for soluble transferrin receptor and retinol-binding protein
Source: PLoS One. 2019 Apr 29;14(4):e0215782. doi: 10.1371/journal.pone.0215782 (PMC6488062; doi:10.1371/journal.pone.0215782)
Supplement: S4 Table — Results in this table can be interpreted as follows: for a future measured value by assay x there is a 95% probability that the future measured value by assay y would be contained in the presented prediction interval. AGP, α-1-acid glycoprotein; CRP, C-reactive protein; Fer, ferritin; RBP, retinol-binding protein; sTfR, soluble transferrin receptor; Roche clinical analyzer assays used as reference assays for Fer, sTfR, CRP, and AGP; retinol measured by HPLC used as reference assay for RBP. (DOCX) [file pone.0215782.s009.docx]

**S4 Table. Predictions and 95% prediction intervals from conversion equations^a^**

|  |  | **Predicted value (95% prediction interval)** |
| --- | --- | --- |
| **Biomarker (unit)** | **Q-Plex™ value** | **Reference** |
| Fer (µg/L) | 6.8 | 11.5 (8.0, 16.4) |
|  | 13.6 | 18.7 (13.0, 26.7) |
|  | 21.7 | 25.9 (18.1, 37.1) |
|  | 44.6 | 42.9 (30.0, 61.4) |
|  | 122 | 86.7 (60.6, 124.1) |
| sTFR (mg/L) | 2.1 | 1.0 (0.7, 1.5) |
|  | 3.8 | 1.8 (1.2, 2.8) |
|  | 4.7 | 2.3 (1.5, 3.4) |
|  | 5.8 | 2.8 (1.9, 4.2) |
|  | 15.9 | 7.7 (5.2, 11.5) |
| CRP (mg/L) | 0.3 | 0.3 (0.2, 0.4) |
|  | 1.0 | 1.0 (0.7, 1.4) |
|  | 2.7 | 2.6 (1.8, 3.8) |
|  | 4.4 | 4.3 (3.0, 6.1) |
|  | 23 | 22.3 (15.6, 32.0) |
| AGP (g/L) | 0.5 | 0.5 (0.4, 0.6) |
|  | 0.7 | 0.7 (0.5, 0.9) |
|  | 0.8 | 0.8 (0.6, 1.1) |
|  | 1.0 | 1.1 (0.8, 1.4) |
|  | 1.5 | 1.6 (1.3, 2.1) |
| RBP (µmol/L) | 0.8 | 0.7 (0.5, 1.1) |
|  | 1.3 | 1.0 (0.7, 1.5) |
|  | 1.6 | 1.2 (0.8, 1.7) |
|  | 1.8 | 1.3 (0.8, 1.9) |
|  | 2.7 | 1.7 (1.1, 2.5) |

^a^ Results in this table can be interpreted as follows: for a future measured value by assay x there is a 95% probability that the future measured value by assay y would be contained in the presented prediction interval. AGP, α-1-acid glycoprotein; CRP, C-reactive protein; Fer, ferritin; RBP, retinol-binding protein; sTfR, soluble transferrin receptor; Roche clinical analyzer assays used as reference assays for Fer, sTfR, CRP, and AGP; retinol measured by HPLC used as reference assay for RBP.
