# Supplementary material for: The Quansys multiplex immunoassay for serum ferritin, C-reactive protein, and α-1-acid glycoprotein showed good comparability with reference-type assays but not for soluble transferrin receptor and retinol-binding protein
Source: PLoS One. 2019 Apr 29;14(4):e0215782. doi: 10.1371/journal.pone.0215782 (PMC6488062; doi:10.1371/journal.pone.0215782)
Supplement: S5 Table — AGP, α-1-acid glycoprotein; CRP, C-reactive protein; Fer, ferritin; RBP, retinol-binding protein; sTfR, soluble transferrin receptor; Roche clinical analyzer assays were used as reference assays for Fer, sTfR, CRP, and AGP; retinol measured by HPLC was used as reference assay for RBP. (DOCX) [file pone.0215782.s010.docx]

**S5 Table.** **Percentage of Q-Plex™ serum sample results that agree with the reference assay results within selected limits^a^**

| **Selected limits** | **Fer** | **sTfR** | **CRP** | **AGP** | **RBP** |
| --- | --- | --- | --- | --- | --- |
| ±5% | 14.1 | 1.20 | 16.7 | 23.5 | 0 |
| ±10% | 26.9 | 1.20 | 48.6 | 51.8 | 2.35 |
| ±15% | 38.5 | 1.20 | 65.3 | 72.9 | 10.6 |
| ±20% | 47.4 | 1.20 | 77.8 | 83.5 | 18.8 |
| ±25% | 53.8 | 2.41 | 84.7 | 89.4 | 25.9 |
| ±30% | 60.3 | 2.41 | 93.0 | 97.6 | 29.4 |

^a^AGP, α-1-acid glycoprotein; CRP, C-reactive protein; Fer, ferritin; RBP, retinol-binding protein; sTfR, soluble transferrin receptor; Roche clinical analyzer assays were used as reference assays for Fer, sTfR, CRP, and AGP; retinol measured by HPLC was used as reference assay for RBP
