# Supplementary material for: The Quansys multiplex immunoassay for serum ferritin, C-reactive protein, and α-1-acid glycoprotein showed good comparability with reference-type assays but not for soluble transferrin receptor and retinol-binding protein
Source: PLoS One. 2019 Apr 29;14(4):e0215782. doi: 10.1371/journal.pone.0215782 (PMC6488062; doi:10.1371/journal.pone.0215782)
Supplement: S6 Table — AGP, α-1-acid glycoprotein; CRP, C-reactive protein; Fer, ferritin; RBP, retinol-binding protein; sTfR, soluble transferrin receptor; 5 serum, heparin plasma, and EDTA plasma samples subjected to up to 5 freeze-thaw cycles (3 h at room temperature/cycle), then analyzed together with the reference samples (no freeze-thaw cycle) in the same run; samples stored at -70°C when not in use. Mean concentration across 5 samples; the SD estimates the variability at the reference condition for each matrix. Percent difference to reference condition was calculated for each sample and then averaged across 5 samples. (DOCX) [file pone.0215782.s011.docx]

**S6 Table. Q-Plex™ freeze-thaw stability of serum, heparin plasma, and EDTA plasma samples^a^**

| **Sample matrix** | **Fer (µg/L)** | **sTfR (mg/L)** | **CRP (mg/L)** | **AGP (g/L)** | **RBP (µmol/L)** |
| --- | --- | --- | --- | --- | --- |
| Serum |  |  |  |  |  |
| Reference condition (SD)^b^ | 47.7 (39.6) | 8.52 (1.71) | 2.55 (1.46) | 0.88 (0.22) | 2.23 (0.52) |
| Mean relative difference to reference condition (95% CI)^c^, % |  |  |  |  |  |
| 1 Freeze/thaw cycle | -0.89 (-5.1, 3.3) | 0.15 (-3.1, 3.4) | -0.60 (-2.5, 13) | 4.1 (1.2, 7.0) | -0.96 (-4.4, 2.5) |
| 2 Freeze/thaw cycles | -1.3 (-8.2, 5.7) | -1.7 (-7.8, 4.3) | 0.52 (-2.7, 3.7) | 8.2 (1.1, 15) | 3.8 (-8.1, 16) |
| 3 Freeze/thaw cycles | -4.4 (-7.9, -0.96) | -0.17 (-4.0, 3.7) | -0.86 (-4.3, 2.6) | 5.6 (0.26, 11) | 1.3 (-4.2, 6.8) |
| 4 Freeze/thaw cycles | -7.1 (-12, -1.7) | -0.72 (-7.5, 6.1) | -2.7 (-6.5, 1.1) | 7.8 (3.6, 12) | 2.4 (-7.9, 13) |
| 5 Freeze/thaw cycles | -1.6 (-5.5, 2.3) | 2.6 (0.80, 4.5) | -2.9 (-9.3, 3.5) | 5.9 (0.17, 12) | 0.51 (-4.3, 5.4) |
| Heparin plasma |  |  |  |  |  |
| Reference condition (SD)^b^ | 45.0 (41.3) | 8.34 (1.58) | 2.68 (1.54) | 0.94 (0.24) | 2.41 (0.44) |
| Mean relative difference to reference condition (95% CI)^c^, % |  |  |  |  |  |
| 1 Freeze/thaw cycle | 3.0 (-3.5, 9.6) | 9.7 (3.2, 16) | -8.1 (-14, -2.4) | -4.8 (-11, 1.5) | -8.6 (-18, 0.35) |
| 2 Freeze/thaw cycles | 7.8 (5.4, 10) | 3.6 (-1.0, 8.2) | -7.9 (-11, -4.9) | -7.1 (-12, -1.7) | -14 (-19, -8.1) |
| 3 Freeze/thaw cycles | 6.7 (2.8, 11) | 1.1 (-1.2, 3.4) | -11 (-16, -5.1) | -7.3 (-15, 0.78) | -17 (-26, -8.2) |
| 4 Freeze/thaw cycles | 13 (5.9, 20) | 4.4 (-1.0, 9.8) | -9.0 (-13, -4.9) | -12 (-15, -9.3) | -14 (-18, -8.5) |
| 5 Freeze/thaw cycles | 15 (5.8, 24) | 6.9 (0.40, 13) | -8.1 (-15, -1.4) | -7.7 (-11, -4.6) | -11 (-20, -1.6) |
| EDTA plasma |  |  |  |  |  |
| Reference condition (SD)^b^ | 48.8 (43.9) | 13.8 (3.37) | 2.54 (1.43) | 0.93 (0.26) | 2.38 (0.44) |
| Mean relative difference to reference condition (95% CI)^c^, % |  |  |  |  |  |
| 1 Freeze/thaw cycle | -2.9 (-7.8, 1.9) | -2.8 (-9.7, 4.1) | -3.8 (-6.2, -1.4) | 0.66 (-1.8, 3.1) | -7.2 (-13, -1.9) |
| 2 Freeze/thaw cycles | 10 (-4.4, 25) | -5.8 (-8.4, -3.1) | -4.0 (-7.6, -0.27) | -9.2 (-16, -2.6) | -14 (-17, -11) |
| 3 Freeze/thaw cycles | 8.1 (-5.7, 22) | -1.5 (-7.6, 4.6) | -4.6 (-6.3, -3.0) | -6.1 (-12, 0.13) | -9.7 (-15, -4.8) |
| 4 Freeze/thaw cycles | 0.68 (-8.2, 9.6) | -1.3 (-4.9, 2.3) | -2.3 (-7.3, 2.8) | -3.2 (-13, 6.2) | -8.4 (-13, -3.5) |
| 5 Freeze/thaw cycles | 1.8 (-6.1, 9.8) | -6.0 (-14, 1.7) | -1.2 (-3.3, 0.85) | -4.0 (-11, 3.4) | -6.0 (-9.4, -2.5) |

^a^ AGP, α-1-acid glycoprotein; CRP, C-reactive protein; Fer, ferritin; RBP, retinol-binding protein; sTfR, soluble transferrin receptor; 5 serum, heparin plasma, and EDTA plasma samples subjected to up to 5 freeze-thaw cycles (3 h at room temperature/cycle), then analyzed together with the reference samples (no freeze-thaw cycle) in the same run; samples stored at -70°C when not in use

^b^ Mean concentration across 5 samples; the SD estimates the variability at the reference condition for each matrix

^c^ Percent difference to reference condition was calculated for each sample and then averaged across 5 samples
