# Supplementary material for: The Quansys multiplex immunoassay for serum ferritin, C-reactive protein, and α-1-acid glycoprotein showed good comparability with reference-type assays but not for soluble transferrin receptor and retinol-binding protein
Source: PLoS One. 2019 Apr 29;14(4):e0215782. doi: 10.1371/journal.pone.0215782 (PMC6488062; doi:10.1371/journal.pone.0215782)
Supplement: S7 Table — AGP, α-1-acid glycoprotein; CRP, C-reactive protein; Fer, ferritin; RBP, retinol-binding protein; sTfR, soluble transferrin receptor. Mean concentration across ~35–40 samples consisting of about 1/3 serum, 1/3 heparin plasma, and 1/3 EDTA plasma samples; the SD estimates the variability at the 2 incubation temperatures. Percent difference to incubation temperature of 18°C was calculated for each sample and then averaged across all samples. Analysis is based on same number of samples analyzed at both incubation temperatures. (DOCX) [file pone.0215782.s012.docx]

**S7 Table. Q-Plex™ results comparing incubation at 30°C with incubation at 18°C^a^**

| **Incubation temperature** | **Fer (µg/L)** | **sTfR (mg/L)** | **CRP (mg/L)** | **AGP (g/L)** | **RBP (µmol/L)** |
| --- | --- | --- | --- | --- | --- |
| Mean concentration (SD)^b^ |  |  |  |  |  |
| 18°C | 90.2 (100) | 10.9 (8.00) | 4.50 (3.10) | 1.09 (0.19) | 3.54 (0.91) |
| 30°C | 86.5 (113) | 23.6 (24.0) | 5.60 (7.01) | 0.668 (0.21) | <0.41 |
| Mean relative percent difference (95% CI)^c^, % | -8.6 (-14, -3.7) | 88 (67, 108) | 5.8 (-5.7, 17) | -39 (-44, -35) | -100 |
| *n*^d^ | 37 | 40 | 35 | 40 | 40 |

^a^ AGP, α-1-acid glycoprotein; CRP, C-reactive protein; Fer, ferritin; RBP, retinol-binding protein; sTfR, soluble transferrin receptor

^b^ Mean concentration across ~35–40 samples consisting of about 1/3 serum, 1/3 heparin plasma, and 1/3 EDTA plasma samples; the SD estimates the variability at the 2 incubation temperatures

^c^ Percent difference to incubation temperature of 18°C was calculated for each sample and then averaged across all samples

^d^ Analysis is based on same number of samples analyzed at both incubation temperatures
